# Validation of transpulmonary thermodilution variables in hemodynamically stable patients with heart diseases

**DOI:** 10.1186/s13613-017-0307-0

**Published:** 2017-08-22

**Authors:** Matthias Peter Hilty, Daniel Peter Franzen, Christophe Wyss, Patric Biaggi, Marco Maggiorini

**Affiliations:** 1 Medical Intensive Care Unit, University Hospital of Zurich, Rämistrasse 100, 8091 Zurich, Switzerland; 2 Department of Pulmonology, University Hospital of Zurich, Zurich, Switzerland; 3 Department of Cardiology, University Hospital of Zurich, Zurich, Switzerland

**Keywords:** Transpulmonary thermodilution, Global end-diastolic volume index, Extravascular lung water index, Cardiac function index, Global ejection fraction, Cardiac disease, Aortic valve stenosis, Dilated cardiomyopathy, Angiography, Pulmonary artery catheter

## Abstract

**Background:**

Transpulmonary thermodilution is recommended in the treatment of critically ill patients presenting with complex shock. However, so far it has not been validated in hemodynamically stable patients with heart disease.

**Methods:**

We assessed the validity of cardiac output, global end-diastolic volume index (GEDVI), an established marker of preload thought to reflect the volume of all four heart chambers, global ejection fraction (GEF) and cardiac function index (CFI) as variables of cardiac function, and extravascular lung water index (EVLWI) as indicator of pulmonary edema in 29 patients undergoing elective left and right heart catheterization including left ventricular angiography with stable coronary heart disease and normal cardiac function (controls, *n* = 11), moderate-to-severe aortic valve stenosis (AS, *n* = 10), or dilated cardiomyopathy (DCM, *n* = 8).

**Results:**

Cardiac output was similar in controls, AS, and DCM, with good correlation between transpulmonary thermodilution and pulmonary artery catheter using the Fick method (*r* = 0.69, *p* < 0.0001). Left ventricular end-diastolic volume was normal in controls and AS, but significantly higher in DCM (104 ± 37 vs 135 ± 63 vs 234 ± 24 ml, *p* < 0.01). GEDVI did not differentiate between patients with normal and patients with enlarged left ventricular end-diastolic volume (848 ± 128 vs 882 ± 213 ml m^−2^, *p* = 0.60). No difference in GEF and CFI was found between patients with normal and patients with reduced left ventricular ejection fraction. Patients with AS but not DCM had higher EVLWI than controls (9 ± 2 vs 12 ± 4 vs 11 ± 3 ml kg^−1^, *p* = 0.04), while there was only a trend in pulmonary artery occlusion pressure (8 ± 3 vs 10 ± 5 vs 14 ± 7 mmHg, *p* = 0.05).

**Conclusions:**

Cardiac output measurement by transpulmonary thermodilution is unaffected by differences in ventricular size and outflow obstruction. However, GEDVI did not identify markedly enlarged left ventricular end-diastolic volumes, and neither GEF nor CFI reflected the increased heart chamber volumes and markedly impaired left ventricular function in patients with DCM. In contrast, EVLWI is probably a sensitive marker of subclinical pulmonary edema particularly in patients with elevated left-ventricular-filling pressure irrespective of differences in left ventricular function.

## Background

Transpulmonary thermodilution is recommended in the management of critically ill patients presenting with complex shock [1, 2], enabling a global assessment of hemodynamic status. Besides measurement of cardiac output, it also provides the volumetric preload parameter global end-diastolic volume index (GEDVI), cardiac function parameters such as global ejection fraction (GEF) and cardiac function index (CFI), and the extravascular lung water index (EVLWI) as a marker of pulmonary edema [3]. In the setting of the critically ill patient in the intensive care unit, GEDVI has been successfully established as an independent marker of cardiac preload in perioperative patients [3] and patients in septic shock [4]. Monnet and co-workers have found that GEDVI tended to be higher in patients with acute heart failure compared with ALI/ARDS patients [5]. Both GEF and CFI have been found to correlate to some extent with ejection fraction as determined by echocardiography in an experimental acute myocardial infarction model [6] and a mixed critically ill patient population [7]. Further, the ratio between EVLWI and GEDVI has been revealed to identify patients with cardiogenic pulmonary edema [5].

Based on these data, the use of transpulmonary thermodilution is generalized across a broad population of critically ill patients, even though the validity of parameters derived from transpulmonary thermodilution remains largely unknown in the setting of cardiac disease. Even though GEDVI is thought to reflect the volume of all four heart chambers [4, 8–10], its validity regarding the distinction between patients with normal and enlarged left ventricles has never been tested. In addition, the validity of GEF and CFI has been tested in hemodynamically unstable patients only, where specific interventions were associated with changes in cardiac output or stroke volume [6, 7, 11]. Again, their validity in hemodynamically stable patients with cardiac disease remains unknown.

In order to fill this important gap, the aim of the present study was to assess the validity of transpulmonary thermodilution-derived flow and volumetric parameters in hemodynamically stable cardiac patients with suspected but stable coronary heart disease (controls), aortic valve stenosis (AS), and dilated cardiomyopathy (DCM) during elective left and right heart catheterization including left ventricular angiography. Our hypothesis was that (I) transpulmonary thermodilution measurement of blood flow is unaffected by differences in ventricular size and outflow obstruction, (II) GEDVI permits the distinction between patients with normal and enlarged left ventricular volumes, (III) GEF and CFI are lower in patients with reduced left ventricular systolic function, and (IV) EVLWI is elevated in patients with left ventricular systolic and diastolic dysfunction.

## Methods

All protocols and procedures conformed to the Declaration of Helsinki and were approved by the local ethics committee (EK-1649). Informed consent for the study protocol and all procedures has been obtained from all patients prior to inclusion. The placement of a pulmonary artery catheter in addition to left heart catheterization has been declared as a study specific intervention in the control group and was clinically mandated in patients with AS and DCM.

### Study population and design

**Fig. 1:**
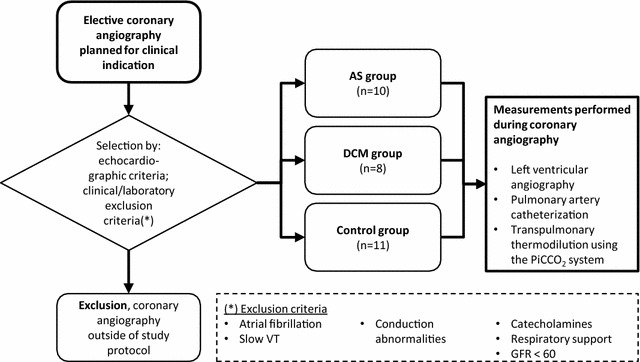
Study design and measurement protocol. Patients planned for elective coronary angiography in a university hospital were included in the study based on the presence of moderate-to-severe aortic valve stenosis or dilated cardiomyopathy in echocardiography. A control group was formed consisting of patients devoid of valvular disease, severe diastolic dysfunction (*E*/*e*′ ratio > 15), and reduced LV-EF <40%. During the coronary angiography session, left and right heart catheterization, left ventricular angiography, and transpulmonary thermodilution were performed. *AS* aortic valve stenosis, *DCM* dilated cardiomyopathy, *LV-EF* left ventricular ejection fraction

Patients planned for elective coronary angiography at the University Hospital of Zurich were screened for eligibility. Clinical examination, thoracic X-ray, serum creatinine analysis, and, in female patients, a pregnancy test were performed. Patients with moderate-to-severe AS and patients with DCM, as determined by echocardiography results, were included (Fig. 1). Patients with suspected coronary artery disease in absence of valvular disease, with preserved left ventricular ejection fraction (LV-EF >40%) [12] and without severe diastolic dysfunction (*E*/*e*′ < 15) [13], were included in the control group. Exclusion criteria were catecholamine-dependent cardiogenic shock, respiratory failure requiring the application of positive end expiratory pressure (due to pulmonary edema), atrial fibrillation, atrioventricular conduction abnormalities, slow ventricular tachycardia, kidney failure (glomerular filtration rate <60 ml min^−1^ [14]), pregnancy, and inability to give informed consent. Included patients underwent elective coronary angiography, during which left ventricular angiography, right heart catheterization, and transpulmonary thermodilution were additionally performed. None of our patients had indirect clinical signs for an aneurysm of the aorta. Directly after angiography, a standardized echocardiography examination was performed. Assignment of patients to the control, AS, and DCM groups was reevaluated based on the echocardiography results obtained during this study. Echocardiography, left and right heart catheter, and transpulmonary thermodilution measurements were compared between the three included patient groups. In addition, patients were divided in two groups of normal or enlarged left ventricular size for comparison of left ventricular end-diastolic volume as determined by left ventricular angiography and GEDVI, as well as two groups of normal or reduced left ventricular function for comparison of left ventricular ejection fraction as determined by left ventricular angiography and GEF and CFI. A left ventricular end-diastolic volume as determined by left ventricular angiography of ≤106 ml in females and ≤150 ml in males and an ejection fraction as determined by left ventricular angiography of >53% in females and >51% in males, were considered normal, respectively [12].

### Left heart catheterization

After puncture of the femoral artery, coronary angiography was performed as clinically indicated and followed by biplanar left ventricular angiography. Aortic pressure (Pa), as well as left ventricular end-diastolic pressure (edPlv), and peak systolic pressure (sPlv) were recorded, and arterial blood was sampled. Left ventricular end-diastolic volume (LV-EDV) and end-systolic volume (LV-ESV) were determined from the left ventricular angiography using digital image analysis software (Xcelera R4.1 build 1173, Philips Medical Systems, Best, the Netherlands) by two independent, blinded examinators. The mean of both results is reported. Stroke volume and LV-EF were calculated from LV-EDV and LV-ESV.

### Right heart catheterization

After puncture of the femoral vein, a 7F balloon-tipped pulmonary artery catheter (Edwards Lifesciences, Irvine, CA, USA) was floated into the pulmonary artery under constant radiologic and pressure wave monitoring for recording of pulmonary artery pressure (Ppa) and pulmonary artery occlusion pressure (Ppao), as well as for mixed venous blood sampling. Then, the catheter was retracted through the right ventricle into the right atrium where right ventricular end-diastolic pressure (edPrv) and right atrial pressure (Pra) were measured. All pressure measurements were recorded at end expiration, and Ppao and Pra were measured at the onset of the c wave that was identified through correlation with the electrocardiogram on the time axis. Blood gas analysis on arterial and mixed venous blood samples for the measurement of arterial and mixed venous hemoglobin oxygen saturation as well as hemoglobin concentration was performed on site without delay (ABL800 Flex, Radiometer, Copenhagen, Denmark). Cardiac output was determined using Fick’s equation using predicted oxygen consumption according to LaFarge and Miettinen [15]. N-terminal B-type natriuretic peptide was determined from a venous blood sample using an electrochemiluminescence immunoassay (Elecsys 2010 analyzer, Roche Diagnostics, Basel, Switzerland) [16].

### Transpulmonary thermodilution measurements

After measurement of Pra, the tip of the Swan-Ganz catheter was carefully positioned at the intersection between the inferior vena cava and the right atrium using fluoroscopy. Transpulmonary thermodilution was then performed using a PiCCO thermodilution catheter inserted through the arterial access sheath and connected to a PiCCO2 monitor (Pulsion Medical Systems, Munich, Germany). Three boli of 20 ml of cold saline tempered to 5 °C were rapidly injected, and in case of an error of >10% in cardiac output between the three measurements, another two measurements were taken. Measurements resulting in a peak temperature difference during the thermodilution bolus of <0.15 °C were discarded. Data from the PiCCO2 monitor were recorded digitally through the system’s USB interface. Parameters directly derived from the thermodilution curve by the monitor’s software included cardiac output (Q), intrathoracic thermal volume (ITTV), and pulmonary thermal volume (PTV), as well as the estimation of intrathoracic blood volume (ITBV) and pulmonary blood volume (PBV). Parameters calculated therefrom included stroke volume (SV), global end-diastolic volume index (GEDVI = [ITTV – PTV]/predicted body surface area), global ejection fraction (GEF = SV/[GEDV/4]), cardiac function index (CFI = Q/GEDV × 10^3^), extravascular lung water index (EVLWI = [ITTV − GEDV × 1.25]/predicted body weight), and pulmonary vascular permeability index (PVPI = EVLW/PBV). In addition, left ventricular stroke work index was calculated using Ppao measured by the pulmonary artery catheter (LVSWI = SV/predicted body surface area × [sPlv − Ppao] × 0.0136). ITTV, PTV, ITBV, and PBV, since they are not reproduced in the PiCCO2 monitor’s digital output, were reverse-calculated from GEDVI and EVLWI as saved by the monitor. The mean of the three-to-five transpulmonary thermodilution measurements is reported for all parameters.

### Echocardiography examination

Echocardiography examinations were performed transthoracically using a stationary device with tissue Doppler imaging capability (iE33 ultrasound system, Koninklijke Philips N.V., Amsterdam, the Netherlands). The left ventricular end-diastolic and end-systolic volume and LV-EF were measured using the biplane disk summation technique [12], in addition to assessment of wall thickness and ventricular mass index. The ratio between mitral inflow velocity and lateral mitral annular velocity (*E*/*e*′) was assessed using pulsed wave and tissue Doppler imaging in order to quantify left ventricular diastolic function. Moderate and severe diastolic dysfunction were considered present given an *E*/*e*′ ratio of 8–15 and >15, respectively [13]. Interpretation of the *E*/*e*′ ratio was omitted in patients with moderate-to-severe mitral regurgitation [13]. Right ventricular fractional area change and tricuspid annular plane systolic excursion were measured to assess right ventricular systolic function. Aortic valve area was estimated using the continuity equation [17]. Moderate and severe AS were considered present with an aortic valve area of 1.0–1.5 and <1.0 mm^2^, respectively [17]. DCM was defined as excentric ventricular hypertrophy with a relative wall thickness ≤0.42 cm and a left ventricular myocardial mass index of >95 and >115 g m^−2^ in females and males, respectively [12, 18]. All measurements were performed in triplicates and recorded digitally for offline assessment which was performed after removal of subject identifiables. The means of the three respective measurements are reported.

### Statistical analysis

Comparisons of measurements between the control, AS, and DCM groups were performed using independent measures one-way analysis of variance (ANOVA), with group membership as the main factor. Pairwise analysis was performed using pairwise two-sample tests with Benjamini and Hochberg’s correction algorithm [19] applied. Comparisons of volumetric measurements between left ventricular angiography and transpulmonary thermodilution were performed using linear correlation employing Pearson’s product-moment correlation coefficient alongside Bland–Altman analysis [20] with percentage error analysis [21]. A two-sided *p* < 0.05 was considered statistically significant. For all statistical analysis, a fully scripted and reproducible data management pathway was created within the R environment for statistical computing, version 3.2.2 [22]. Graphical output was generated using the R library ggplot2, version 2.0.0 [23]. Values are given as mean ± standard deviation (SD).

## Results

### Patient characteristics

Twenty-nine patients were included, 10 with AS, 8 with DCM, and 11 in the control group. Patients with AS were older than those with DCM and controls (Table 1). All patients had similar body surface area and hemoglobin concentration. Patients in the AS and DCM groups were fairly well compensated with a mean NYHA class between 2 and 3. Only one patient in the AS and DCM groups, respectively, demonstrated rales on clinical lung examination, and one patient in the DCM group had signs of redistribution on the thoracic X-ray film. Patients in the DCM group presented with a slightly higher lactate and N-terminal B-type natriuretic peptide levels than patients with AS or controls. In all groups, mean lactate concentration was less than 2 mmol l^−1^. Echocardiography indicated severe AS in 4/10 (40%) patients in the AS group and moderate AS in the remaining six. Overall, the mean aortic valve area was 1.0 mm^2^, and the mean pressure gradient was 26 mmHg in the AS group (Table 1). An increasing left ventricular mass index was observed starting from the control group with progression to the AS and DCM groups, while the DCM group presented with enlarged LV-EDV. The latter patients presented with a decreased LV-EF, while all groups demonstrated similar diastolic function as measured by *E*/*e*′, with a trend toward higher values in the AS group. Ppao was 14 mmHg in the DCM group, whereas it was 10 and 8 mmHg in the AS group and controls, respectively (*p* = 0.05). Three patients in the DCM group suffered from moderate-to-severe secondary mitral regurgitation, as opposed to none in the AS and control groups. Patients in the DCM group also presented with only slightly increased right ventricular longitudinal diameter as compared to the AS and control groups, with similar right ventricular transversal diameters. Right ventricular function and Pra were normal in all groups. No patients presented with moderate-to-severe tricuspid regurgitation. Pulmonary vascular resistance was normal in all groups.

**Table 1:** Patient characteristics

	Control *n* = 11	AS *n* = 10	DCM *n* = 8	*p*
Age [a]	62 ± 7	78 ± 11^a^	56 ± 14^b^	<0.001
Sex (male)	9/11	7/10	7/8	0.64
BSA predicted [m^2^]	1.80 ± 0.15	1.74 ± 0.16	1.82 ± 0.11	0.46
NYHA class [1]	1.5 ± 0.5	2.4 ± 0.7	2.0 ± 1.2	0.07
Hb [g l^−1^]	139 ± 18	133 ± 9	132 ± 15	0.51
Lactate [mmol l^−1^]	0.8 ± 0.3	0.5 ± 0.2	0.9 ± 0.3^b^	0.02
N-terminal B-type natriuretic peptide [ng l^−1^]	177 ± 216	882 ± 994	1423 ± 1132^a^	0.01
Echocardiography, left ventricle (LV)				
LV end-diastolic volume [ml]	98 ± 35	97 ± 21	181 ± 25^ab^	<0.0001
LV myocardial mass index [g m^−2^]	96 ± 16	116 ± 21^a^	145 ± 19^ab^	<0.001
LV ejection fraction [%]	64 ± 7	60 ± 13	32 ± 9^ab^	<0.0001
*E*/*e*′ ratio [1]	9.4 ± 3.3	12.0 ± 2.3	11.0 ± 3.4^(*)^	0.21
Echocardiography, right ventricle (RV)				
RV transversal diameter (D2) [mm]	27 ± 6	26 ± 5	29 ± 11	0.73
RV longitudinal diameter (D3) [mm]	68 ± 5	66 ± 10	78 ± 12^b^	0.04
Tricuspid annular plane systolic excursion [mm]	23 ± 4	23 ± 6	19 ± 5	0.23
RV fractional area change [%]	47 ± 11	52 ± 12	50 ± 17	0.74
Echocardiography, aortic valve				
Aortic valve area [mm^2^]	3.0 ± 1.5	1.0 ± 0.4^a^	2.5 ± 0.4^b^	<0.0001
Aortic valve peak instantaneous pressure gradient [mmHg]	3.8 ± 3.4	48.9 ± 26.2^a^	5.4 ± 2.2^b^	<0.0001
Aortic valve mean pressure gradient [mmHg]	2.3 ± 2.1	26.2 ± 17.8^a^	3.4 ± 1.2^b^	<0.001
Echocardiography, mitral valve				
Secondary, moderate-to-severe mitral regurgitation	0/11	0/10	3/8	0.07

Values are given as mean ± SD. Pairwise analysis is represented by^a,b^ where *p* < 0.05 versus the control and AS groups, respectively. ^(*)^ *n* = 5 for *E*/*e*′ ratio reported in the DCM group, since *E*/*e*′ ratio measurements were discarded in patients with moderate-to-severe mitral regurgitation

*AS* aortic valve stenosis, *DCM* dilated cardiomyopathy, *BSA* body surface area, *NYHA* New York Heart Association functional classification of heart failure, *Hb* hemoglobin

### Cardiac output and contractility

**Fig. 2:**
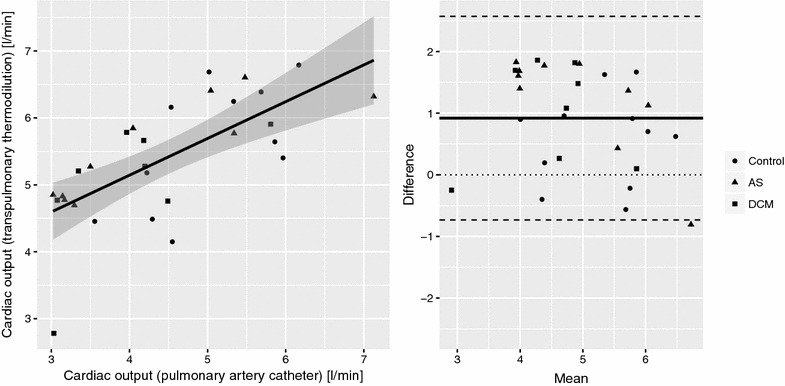
Good correlation between cardiac output measurements by transpulmonary thermodilution and pulmonary artery catheter was observed [*r* = 0.69, *p* < 0.0001, bias 0.9 l min^−1^ (extended black line in the Bland-Altman plot), precision 0.8 l min^−1^, level of agreement −0.7 to 2.6 l min^−1^ (dashed black line)]. The shaded area in the linear correlation plot denotes the fitted linear model’s 95% confidence interval. *AS* aortic valve stenosis, *DCM* dilated cardiomyopathy

**Fig. 3:**
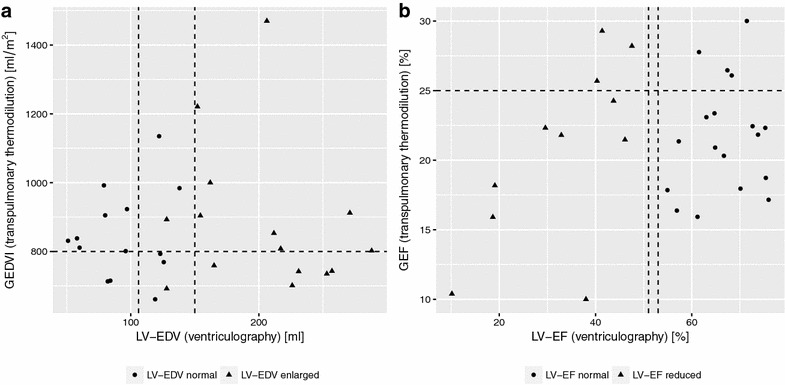
Comparison of volumetric parameters and measures of contractility as determined by left ventricular angiography and transpulmonary thermodilution. Patients with normal versus enlarged left ventricular end-diastolic volume presented with similar values of GEDVI (**a**). Similarly, GEF did not differentiate patients with normal versus reduced left ventricular ejection fraction (**b**). *Vertical dashed lines* represent cutoff values for the normal range of left ventricular end-diastolic volume (**a**) and left ventricular ejection fraction (**b**) in females and males, respectively. *Horizontal dashed lines* represent the upper normal range for GEDVI (**a**) and lower normal range for GEF (**b**). *GEDVI* global end-diastolic volume index, *GEF* global ejection fraction

As assessed by transpulmonary thermodilution, cardiac output and stroke volume were within normal range and similar between controls, AS and DCM groups (Table 2), despite a significantly lower LV-EF (32%) in the latter group as compared to LV-EF > 55% in the AS and control groups (*p* < 0.001; Tables 1 and 2) as determined by left ventricular angiography as well as echocardiography. A good correlation between cardiac output measurements by transpulmonary thermodilution and pulmonary artery catheter via the Fick method was observed (*r* = 0.69, *p* < 0.0001). Bland–Altman analysis revealed a bias of 0.9 l min^−1^ with a precision 0.8 l min^−1^ (level of agreement −0.7 to 2.6 l min^−1^) and mean percentage error of 33% (Fig. 2). No difference in GEF and CFI was found between the three groups (Table 2), as well as between patients with normal versus reduced LV-EF as determined by left ventricular angiography regardless of group allocation (Table 3; Fig. 3b). In addition, there was no linear relationship between LV-EF as measured by left ventricular angiography and GEF (*r* = 0.32, *p* = 0.09) or CFI (*r* = 0.27, *p* = 0.15) as measured by transpulmonary thermodilution. In contrast, LVSWI was significantly lower in DCM patients when compared to the other two groups (Table 2).

**Table 2:** Hemodynamic status and transpulmonary thermodilution measurements in patients with aortic valve stenosis, dilated cardiomyopathy, and controls

	Control *n* = 11	AS *n* = 10	DCM *n* = 8	*p*
Heart rate [min^−1^]	70 ± 9	67 ± 6	71 ± 13	0.62
Cardiac output (pulmonary artery catheter) [l min^−1^]	5.0 ± 0.9	4.3 ± 1.4	4.0 ± 0.9	0.13
Left ventricular peak systolic pressure (sPlv) [mmHg]	145 ± 29	160 ± 34	126 ± 19	0.06
Mean aortic pressure (mPa) [mmHg]	103 ± 20	91 ± 14	90 ± 12	0.15
Left ventricular stroke work index (LVSWI) [g m s^−2^ m^−2^]	82 ± 12	100 ± 28	63 ± 23^b^	<0.01
Transaortic peak-to-peak pressure gradient (sPlv–sPa) [mmHg]	1 ± 8	19 ± 14^a^	1 ± 4^b^	<0.001
Right ventricular end-diastolic pressure (edPrv) [mmHg]	6 ± 4	6 ± 6	9 ± 6	0.27
Right atrial pressure (Pra) [mmHg]	4 ± 3	4 ± 2	7 ± 5	0.14
Mean pulmonary artery pressure (mPpa) [mmHg]	16 ± 6	16 ± 7	24 ± 11	0.08
Pulmonary artery occlusion pressure (Ppao) [mmHg]	8 ± 3	10 ± 5	14 ± 7^a^	0.05
Transpulmonary pressure gradient (mPpa–Ppao) [mmHg]	8 ± 4	6 ± 3	9 ± 6	0.38
Pulmonary vascular resistance [dyn s cm^−5^]	108 ± 55	93 ± 50	173 ± 174	0.23
Biplanar left ventricular angiography				
Stroke volume [ml]	64 ± 26	77 ± 20	76 ± 32	0.46
Left ventricular end-diastolic volume [ml]	104 ± 37	135 ± 63	234 ± 24^ab^	<0.0001
Left ventricular ejection fraction [%]	62 ± 11	63 ± 16	32 ± 12^ab^	<0.0001
Transpulmonary thermodilution (PiCCO2)				
Cardiac output [l min^−1^]	5.6 ± 0.9	5.5 ± 0.7	5.0 ± 1.0	0.35
Stroke volume [ml]	81 ± 15	84 ± 15	75 ± 25	0.57
Global end-diastolic volume index (GEDVI) [ml m^−^^2^]	823 ± 106	909 ± 170	870 ± 252	0.54
Global ejection fraction (GEF) [%]	22 ± 4	22 ± 4	20 ± 7	0.54
Cardiac function index (CFI) [min^−1^]	3.9 ± 0.8	3.6 ± 0.7	3.3 ± 0.8	0.32
Extravascular lung water index (EVLWI) [ml kg^−1^]	9 ± 2	12 ± 4^a^	11 ± 3	0.04
Pulmonary vascular permeability index (PVPI) [1]	2 ± 0	2 ± 0	2 ± 1	0.12

Values are given as mean ± SD. Pairwise analysis is represented by^a,b^ where *p* < 0.05 versus the control and AS groups, respectively

*AS* aortic valve stenosis, *DCM* dilated cardiomyopathy

**Table 3:** Comparison of volumetric parameters and measures of contractility as determined by left ventricular angiography and transpulmonary thermodilution

	Normal left ventricular end-diastolic volume *n* = 14	Enlarged left ventricular end-diastolic volume *n* = 15	p
Left ventricular end-diastolic volume [ml] (left ventricular angiography)	94 ± 28	203 ± 52	<0.0001
Global end-diastolic volume index (GEDVI) [ml m^−2^]	848 ± 128	882 ± 213	0.60

	Normal left ventricular ejection fraction *n* = 18	Reduced left ventricular ejection fraction *n* = 11	
Left ventricular ejection fraction [%] (left ventricular angiography)	67 ± 7	33 ± 13	<0.0001
Global ejection fraction (GEF) [%]	22 ± 4	21 ± 6	0.66
Cardiac function index (CFI) [min^−1^]	3.7 ± 0.7	3.5 ± 0.9	0.61

Values are given as mean ± SD

### Cardiac volumes

Patients in the DCM group were well differentiated from the AS and control groups in left ventricular angiography by their larger LV-EDV (*p* < 0.001 in pairwise analysis for DCM versus AS and DCM versus controls). No difference was found in GEDVI between all three groups (Table 2), as well as between patients with normal versus enlarged left ventricular end-diastolic volume as determined by left ventricular angiography (Table 3; Fig. 3a). Similarly, neither the mean transit time or the exponential downslope time derived from the transpulmonary thermodilution curves, nor the intrathoracic or the pulmonary thermal volume differed between the three groups (Table 4). Further, no linear relationship was found between LV-EDV indexed for predicted body surface area as measured by left ventricular angiography and GEDVI as measured by transpulmonary thermodilution (*r* = 0.002, *p* = 0.99).

**Table 4:** Transpulmonary thermodilution mathematical model parameters

	Control *n* = 11	AS *n* = 10	DCM *n* = 8	p
Mean transit time (MTt) [s]	442.9 ± 91.0	497.9 ± 88.7	570.8 ± 199.2	0.12
Exponential downslope time (DSt) [s]	173.1 ± 41.1	211.2 ± 47.2	246.0 ± 107.0	0.08
Thermal indicator volumetric parameters				
Intrathoracic thermovolume (ITTV) [ml]	2439 ± 470	2759 ± 662	2744 ± 754	0.43
Pulmonary thermovolume (PTV) [ml]	956 ± 230	1176 ± 355	1159 ± 320	0.20
Estimated dye indicator volumetric parameters				
Intrathoracic blood volume (ITBV) [ml]	1854 ± 327	1979 ± 441	1982 ± 594	0.77
Pulmonary blood volume (PBV) [ml]	371 ± 65	396 ± 88	396 ± 119	0.77

Values are given as mean ± SD

*AS* aortic valve stenosis, *DCM* dilated cardiomyopathy

### Extravascular lung water

**Fig. 4:**
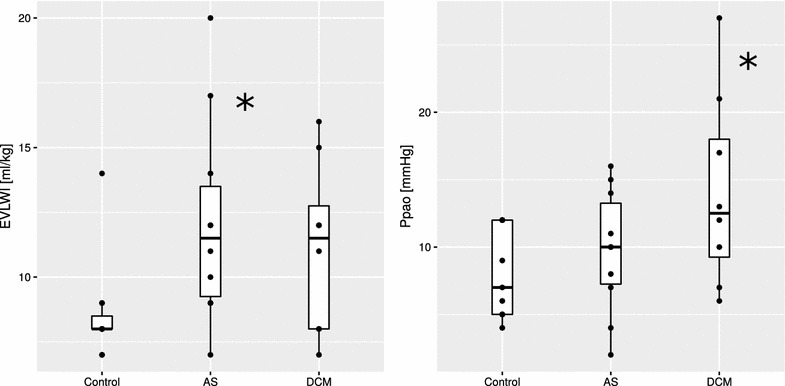
Comparison of extravascular lung water index (EVLWI) as measured by transpulmonary thermodilution and pulmonary artery occlusion pressure (Ppao). In patients with aortic valve stenosis and dilated cardiomyopathy a trend toward increased EVLWI and Ppao was detected, respectively, that may be related to subclinical pulmonary edema. ANOVA *p* = 0.04 for EVLWI, (*) denotes a significant difference versus the control group in pairwise analysis (*p* < 0.05). *AS* aortic valve stenosis, *DCM* dilated cardiomyopathy, *ANOVA* one-way analysis of variance

All ten of the patients in the AS group presented with normal LV-EF but moderate-to-severe diastolic dysfunction. In the DCM group, all patients presented with significantly depressed LV-EF, but only 6 of 8 (75%) with moderate-to-severe diastolic dysfunction. As selected by the inclusion criteria, in the control group none of the patients presented with severe diastolic dysfunction, but in 5/11 (45%) moderate diastolic dysfunction was present. Overall *E*/*e*′ ratio in the AS, DCM, and control groups was similar, with a trend toward higher mean *E*/*e*′ ratio in the AS group (Table 1). Ppao was highest in DCM (14 mmHg), but normal (≤10 mmHg) in the other two groups (*p* = 0.05, Table 2; Fig. 4). Patients with AS but not DCM had higher EVLWI than controls (*p* < 0.05 in pairwise analysis for DCM vs. control, Fig. 4), a signal that remained undetected by raw thermodilution data (Table 4) before mathematical processing was applied in order to derive EVLWI, namely subtracting a multiple of the difference between ITTV and PTV from ITTV itself. Pulmonary vascular permeability index (PVPI) was found to be <3 in all groups. Ppao as measured by pulmonary artery catheter correlated well to edPlv as measured by left ventricular angiography (*r* = 0.63, *p* < 0.001; bias −8 mmHg, precision 5 mmHg, level of agreement −19 to 3 mmHg), while no linear relationship was found between EVLWI and Ppao (*r* = 0.10, *p* = 0.60).

## Discussion

The present study, using state-of-the art invasive reference hemodynamic measurements, demonstrates that (I) transpulmonary thermodilution measurement of blood flow is unaffected by differences in ventricular size and outflow obstruction. However, (II) GEDVI as measured by transpulmonary thermodilution does not differentiate between patients with a normal-sized left ventricle and those with a left ventricle enlarged by DCM. This finding may not contradict GEDVI’s role as a preload indicator, but it might require a revision of its understanding as the volume of the four heart chambers. Furthermore, in our hemodynamically stable patients, (III) both transpulmonary thermodilution-derived cardiac function parameters, GEF and CFI, failed to detect impaired LV-EF in DCM patients. In addition, we found that (IV) EVLWI is a sensitive marker for the detection of subclinical pulmonary edema in patients with impaired diastolic and systolic cardiac function.

### Cardiac output

Blood flow measurements by thermodilution are a specialized case of the indicator dilution principle based on a slightly modified Stewart–Hamilton formula [24]. The thermodilution technique has been successfully applied to measure regional blood flow in single blood vessel [25] as well as across a pulmonary artery catheter in order to assess global cardiac output [26]. Obviating the need for pulmonary artery catheterization by injection of a bolus of cold saline into the superior or inferior vena cava that is transported with the blood flow and detected by a thermistor inserted into the femoral artery, transpulmonary thermodilution has been proven to match thermodilution measurement of blood flow across the right ventricle using a pulmonary artery catheter in a perioperative setting [27, 28], in patients with heart failure primarily due to coronary heart disease [11] and in patients with severe mitral regurgitation undergoing valve repair [29]. The measurement of cardiac output has further been validated against echocardiography in patients with severe AS [30]. Our study confirms these findings and furthermore expands the results to patients with DCM, presenting with severely enlarged left ventricular dimensions and moderately to severely impaired LV-EF, in a comparison with pulmonary artery catheter measurements. The finding allows the conclusion that turbulences in regional blood flow as they can be induced by obstruction at a stenosed aortic valve as well as by the presence of an enlarged left ventricle in the indicator’s path, do not influence the result of the Stewart–Hamilton equation to a clinically relevant extent. Therefore, measurement of cardiac output in this setting is reliable.

### Global end-diastolic volume index

In analogy to flow measurements by transpulmonary indicator dilution, volumetric measurements have been developed using dye dilution. Some of these findings have later been translated to the use of a thermal indicator. Early findings suggest that the volume in-between the injection and detection sites minus the largest volume chamber in the series may describe the combined volume of all four heart chambers [31, 32], temporally averaged over the measurement cycle of the indicator’s first pass. This new entity has been termed global end-diastolic volume. GEDVI is defined as the difference between the ITTV and the PTV, divided by the predicted body surface area [3]. ITTV represents the distribution volume of the thermal indicator as determined by the product of cardiac output and the mean transit time (MTt) of the indicator bolus [32], and PTV is the product of cardiac output and the reciprocal of the elimination constant of the first-order elimination (exponential downslope time, DSt) of the indicator during its first pass through the measurement site (before recirculation occurs). It reflects the largest volume chamber in series between the site of injection and the tip of the femoral catheter [31]. Notably, the interconnection between DSt and volume chambers serially passed by an indicator has been discovered and validated by Newman and co-workers in an in vitro model circulation rather than the human heart. Derived parameters such as GEDVI ultimately reflect the dissipation of an indicator through the volume chambers of the thoracic cavity. Even though GEDVI is thought to reflect the volume of all four heart chambers [31, 32], what it represents in a physiological context is less clear. Nevertheless, in the setting of a hemodynamically unstable critically ill or perioperative patient, GEDVI has been found to be an independent predictor of cardiac preload [3, 4]. GEDVI has previously been found to correlate to LV-EDV as measured by echocardiography in a population of patients undergoing elective coronary artery bypass surgery [9, 33], with ventricular function and volumes close to normal. GEDVI’s relatively high inter-individual variability has been partially attributed to age and gender differences [34]. The present study for the first time adds to this understanding that no such association seems to exist in a patient population selected for large differences in left ventricular end-diastolic volume, by demonstrating that neither the direct properties of the transpulmonary thermodilution curve such as MTt and DSt, nor the derived parameters, ITTV, PTV, and GEDVI, contain a signal that differentiates between patients with normal versus enlarged LV-EDV. This holds true even though the patients presenting with enlarged LV-EDV also had slightly enlarged right ventricles. Confounding factors were minimal, since all patients included in the present study had normal right ventricular and tricuspid valve function, the catheter tip was accurately placed at the entry of the right atrium by fluoroscopy and the presence of aortic aneurysms had been excluded using angiography. In addition to these factors, due to the nature of transpulmonary thermodilution, GEDVI is averaged over the complete cardiac cycle and the term “end-diastolic” seems to be an oversimplification first appearing in the work by Goedje and co-workers [3]. Our findings may thus change our perception of GEDVI in a physiological context. GEDVI should be understood as a parameter resulting from the suppression of the signal of pulmonary blood volume and extravascular lung water from the transpulmonary thermodilution curve, without differentiating between the change in volume that occurs during the cardiac cycle. What remains is information about cardiac preload, but not effective left ventricular end-diastolic volume. The use of ITBV as the indicator of preload would avoid misunderstanding in the future.

### Cardiac function

The CFI has been conceived as a parameter reflecting cardiac performance independently of changes in intrathoracic pressure, myocardial compliance, and vascular tone [35]. GEF is another cardiac functional parameter which is independent of heart rate. In analogy to the ventricular ejection fraction as measured by left ventricular angiography or echocardiography, GEF is defined as the quotient between the stroke volume and global end-diastolic volume divided by the number of the cardiac cavities (4). In pigs, good correlations were demonstrated between both CFI and GEF and left ventricular dP/dt [35, 36] and ejection fraction as determined by echocardiography in acute myocardial infarction [4]. Similar correlations were found in a mixed critically ill patient population between LV-EF as determined by echocardiography and both CFI and GEF [7, 8]. In all these studies, changes in CFI and/or GEF were associated with changes in cardiac output. In patients with septic shock, Jabot and co-workers found that GEF and CFI reflect the application of positive inotropy but not a volume challenge [37]. Ritter and co-workers, comparing patients with septic shock and acute heart failure, found CFI and GEF to be significantly lower in acute heart failure patients [11]. In this study, cardiac output was 4.6 l min^−1^ in septic and 2.7 l min^−1^ in acute heart failure patients, whereas GEDVI was not different between groups (907 ml in sepsis and 995 ml in acute heart failure). In contrast, in our hemodynamically stable patients with no significant differences in cardiac output, stroke volume, and GEDVI, CFI and GEF did not reveal markedly depressed LV-EF of the DCM patients. However, calculating LVSWI—which takes into account besides stroke volume also the difference between mean Pa and Ppao—significant differences were found between the DCM group and the other two. Thus, our data suggest that CFI and GEF values are surrogate markers of global cardiac function but not LV-EF, hence cardiac contractility. In euvolemic hemodynamically stable patients monitored with a pulmonary artery catheter, calculation of LVSWI may be more suitable for the assessment of cardiac power, as Ppao values reflect myocardial systolic and diastolic function.

### Extravascular lung water

Due to thermodynamic as well as hemodynamic interactions within the pulmonary capillary network, the signal derived from transpulmonary thermodilution also contains information about extravascular lung water that can be extracted by referencing it with a concomitantly performed inert dye dilution [38]. EVLWI is thus defined as the difference between ITBV and ITTV divided by predicted body weight. ITBV is determined by dye dilution analogous to how ITTV is determined by thermodilution. Based on a largely linear relationship between global end-diastolic volume as determined by single-indicator transpulmonary thermodilution and ITBV as determined by single-indicator transpulmonary dye dilution as described in human studies [8, 39], ITBV is approximated by the PiCCO2 monitor by extrapolating global end-diastolic volume using a constant factor of 1.25. EVLWI calculated in this way is thought to reflect the extravascular water content of the lung within the same physiological analogy that connects GEDVI with the volume of all four heart chambers. It has been demonstrated to reliably detect the presence of pulmonary edema [40] and through division by PBV, yielding PVPI, enable the differentiation of its etiology [5]. It has also been suggested as a predictor for ICU mortality in a mixed ICU population [41]. Our study revealed an increased EVLWI in patients with moderate-to-severe AS and to a lesser extent in DCM. These results are consistent with previous observations showing that EVLWI may be elevated in hemodynamically stable patients suffering from heart failure with reduced LV-EF (≤40%) as compared to healthy controls [42]. In our study population, AS patients presented with preserved LV-EF and overall only moderate-to-severe AS and are thus expected to tolerate higher physical performance levels as compared to the DCM patients. The finding that EVLWI values were highest in AS patients could hence be explained by a very high Ppao during physical activity, leading to exercise induced subclinical pulmonary edema. Since interstitial lung water may take one or more days to clear, while Ppao has already returned to baseline [43], EVLWI could indeed be an earlier indicator of continually or intermittently elevated pulmonary capillary pressure due to compromised systolic and/or diastolic cardiac function. This argument is further supported by our finding of elevated N-terminal B-type natriuretic peptide levels in patients with DCM with a trend toward higher values in the AS group, while clinical and radiological signs of pulmonary edema or pleural effusion were absent in all groups. Additionally, the lack of correlation between Ppao and EVLWI in our study adds to previous observations in mixed patient populations [11, 43]. In our study, Ppao measurements represent a single observation in time while patients were lying supine and at rest.

### Limitations

The present study was designed to compare transpulmonary thermodilution measurements to left ventricular angiography and pulmonary artery catheterization in hemodynamically stable patients with cardiac disease. Our data only encompass one point in time, and the examinations were not repeated during the course of treatment. Thus, no conclusions can be made regarding the dynamic response of the examined parameters to interventions. Additionally, even though left ventricular angiography is the accepted gold standard used to determine ventricular end-systolic and end-diastolic volumes, there are several technical limitations. A similar disk summation algorithm [12] is applied to the biplanar image of the ventricle as is commonly employed in echocardiography, but signal quality achieved by angiography is enhanced as compared to echocardiography due to increased spatial resolution and the possibility to analyze both perpendicular planes within the same heartbeat. Still, some error remains through the reliance on a biplanar instead of a three-dimensional capture. While the close correlation of the values to measurements by echocardiography supports the validity of the data assessed in this study, it is not possible to derive information about the magnitude of this error, although it is expected to be small [12]. Further, while in the present study, measurement of stroke volume by thermodilution has been found to be accurate as compared to left ventricular angiography in obstructive and dilated heart disease, a conclusion cannot be reached regarding valve regurgitation based on the data presented. In the case of valve regurgitation, oscillating blood volume violates additional prerequisites for the Stewart–Hamilton formula as compared to obstructive and dilated heart disease. In the present study, the subgroup of patients exhibiting moderate-to-severe mitral regurgitation is not large enough to derive a conclusion. Further studies are warranted to answer this question.

## Conclusions

Cardiac output measurement by transpulmonary thermodilution is unaffected by outflow obstruction in aortic valve stenosis and large differences in left ventricular end-diastolic volume as present in dilated cardiomyopathy. However, in our study, GEDVI did not identify markedly enlarged left ventricular end-diastolic volumes in patients with dilated cardiomyopathy. In addition, neither GEF nor CFI reflected markedly reduced left ventricular ejection fraction in the same patient population. Thus, in hemodynamically stable cardiac patients, transpulmonary thermodilution has clear limitations and needs to be interpreted with caution. In contrast, EVLWI is probably a sensitive marker of subclinical pulmonary edema, particularly in patients with aortic valve stenosis presenting with elevated left ventricular filling pressure, irrespective of differences in left ventricular function.

## Abbreviations

AS: aortic valve stenosis
CFI: cardiac function index
DCM: dilated cardiomyopathy
DSt: exponential downslope time
EVLWI: extravascular lung water index
GEDVI: global end-diastolic volume index
GEF: global ejection fraction
ITBV: intrathoracic blood volume
ITTV: intrathoracic thermal volume
LV-EDV: end-diastolic left ventricular volume
LV-EF: left ventricular ejection fraction
LV-ESV: end-systolic left ventricular volume
LVSWI: left ventricular stroke work index
MTt: mean transit time
Pa: aortic pressure
Plv: left ventricular pressure
Ppao: pulmonary artery occlusion pressure
Pra: right atrial pressure
PBV: pulmonary blood volume
PTV: pulmonary thermal volume
PVPI: pulmonary vascular permeability index
